# A new mutation of *Sgms1* causes gradual hearing loss associated with a reduced endocochlear potential

**DOI:** 10.1016/j.heares.2024.109091

**Published:** 2024-07-23

**Authors:** Jing Chen, Morag A Lewis, Alisa Wai, Lucia Yin, Sally J Dawson, Neil J Ingham, Karen P Steel

**Affiliations:** a Wolfson Sensory, Pain and Regeneration Centre, King’s College London, London SE1 1UL, United Kingdom; b UCL Ear Institute, University College London, London WC1X 8EE, United Kingdom

**Keywords:** Sgms1, Progressive hearing loss, Stria vascularis, Endocochlear potential, Sphingosine-1-phosphate signalling

## Abstract

*Sgms1* encodes sphingomyelin synthase 1, an enzyme in the sphingosine-1-phosphate signalling pathway, and was previously reported to underlie hearing impairment in the mouse. A new mouse allele, *Sgms1*^*tm1a*^, unexpectedly showed normal Auditory Brainstem Response thresholds. We found that the *Sgms1*^*tm1a*^ mutation led to incomplete knockdown of transcript to 20 % of normal values, which was enough to support normal hearing. The *Sgms1*^*tm1b*^ allele was generated by knocking out exon 7, leading to a complete lack of detectable transcript in the inner ear. *Sgms1*^*tm1b*^ homozygotes showed largely normal auditory brainstem response thresholds at first, followed by progressive loss of sensitivity until they showed severe impairment at 6 months old. The endocochlear potential was consistently reduced in *Sgms1*^*tm1b*^ mutants at 3, 4 and 8 weeks old, to around 80 mV compared with around 120 mV in control littermates. The stria vascularis showed a characteristic irregularity of marginal cell surfaces and patchy loss of Kcnq1 expression at their apical membrane, and expression analysis of the lateral wall suggested that marginal cells were the most likely initial site of dysfunction in the mutants. Finally, significant association of auditory thresholds with DNA markers within and close to the human *SGMS1* gene were found in the 1958 Birth Cohort, suggesting that *SGMS1* variants may play a role in the range of hearing abilities in the human population.

## 1 Introduction

Age-related, progressive hearing loss is a common phenomenon in the population and can have a significant impact upon the quality of life, including increased susceptibility to social isolation, depression and dementia (Livingston et al., 2020; Fellinger et al., 2012; Karpa et al., 2010; Mick et al., 2014; Livingston et al., 2017). However, little is known about the molecular mechanisms underlying age-related hearing loss. We previously carried out a large-scale screen of newly-generated mouse mutants to search for new genes underlying progressive hearing loss using Auditory Brainstem Response (ABR) recording (Ingham et al., 2019). Surprisingly one of the known deafness genes targeted, *Sgms1*, did not show raised ABR thresholds at the age screened, 14 weeks old. The mouse *Sgms1*^*tm1a*^ mutation that was screened was reported to lead to a range of phenotypes including male infertility, decreased body fat, reduced platelet count and increased platelet volume (https://www.mousephenotype.org/data/genes/MGI:2444110; Birling et al., 2021; White et al., 2013), as well as small lateral brain ventricles (Collins et al., 2019), so the lack of any effect of the mutation on ABR thresholds was not because of a complete lack of impact of the mutation. A previous paper described a different mouse *Sgms1* mutant with hearing loss (Lu et al., 2012), so we followed up the new mutant allele to investigate its role in hearing.

*Sgms1* encodes sphingomyelin synthase 1, an enzyme in the sphingosine-1-phosphate (S1P) signalling pathway. It regulates the synthesis of sphingomyelin from ceramide and phosphatidylcholine, producing diacylglycerol in the process. Ceramide alternatively can be converted to sphingosine which in turn can be phosphorylated to S1P, an active signalling molecule. S1P acts as a ligand for five G-protein coupled receptors, S1P receptors 1–5, with downstream effects on a range of cellular processes. The sphingosine-1-phosphate signalling pathway is known to be involved in hearing as mutations in the S1P transporter gene *Spns2* and the S1P receptor gene *S1pr2* lead to deafness (Chen et al., 2014; Herr et al., 2007; Kono et al., 2007; MacLennan et al., 2006; Ingham et al., 2016; Santos-Cortez et al., 2016; Ingham et al., 2019; Hofrichter et al., 2018; Mardani et al., 2023).

The *Sgms1*^*tm1a*^ allele has a large DNA cassette inserted in an intron intended to disrupt transcription of the gene. Our analysis of the *Sgms1*^*tm1a*^ mutation indicated that there was an incomplete knockdown of the mRNA levels and suggested that 20% of its usual expression level is adequate for normal auditory responses. We generated the *Sgms1*^*tm1b*^ allele by deleting exon 7 from *Sgms1*^*tm1a*^, and found these homozygous mutants did show slowly progressive hearing loss associated with a non-progressive reduction in endocochlear potential.

## 2 Methods

### 2.1 Ethics statement

Mouse studies were carried out in accordance with UK Home Office regulations and the UK Animals (Scientific Procedures) Act of 1986 under UK Home Office licences, and the study was approved by the King’s College London Animal Welfare and Ethical Review Body. Mice were culled using methods approved under these licences to minimise any possibility of suffering. Mice were group-housed in individually-ventilated cages at a standard temperature and humidity and in specific-pathogen-free conditions, with lighting on a 12 h on/12 h off cycle, and in accordance with the EU Directive 2010/63/EU for animal experiments. Both males and females were used in all experiments and there were no apparent differences between the sexes except where stated (weights). Experiments were carried out between 2 and 10 h after lights on except for expression studies when samples were collected within a 1.5 hour window from 6 h after lights on.

### 2.2 Production and genotyping of Sgms1 mutant mice

**Fig. 1:**
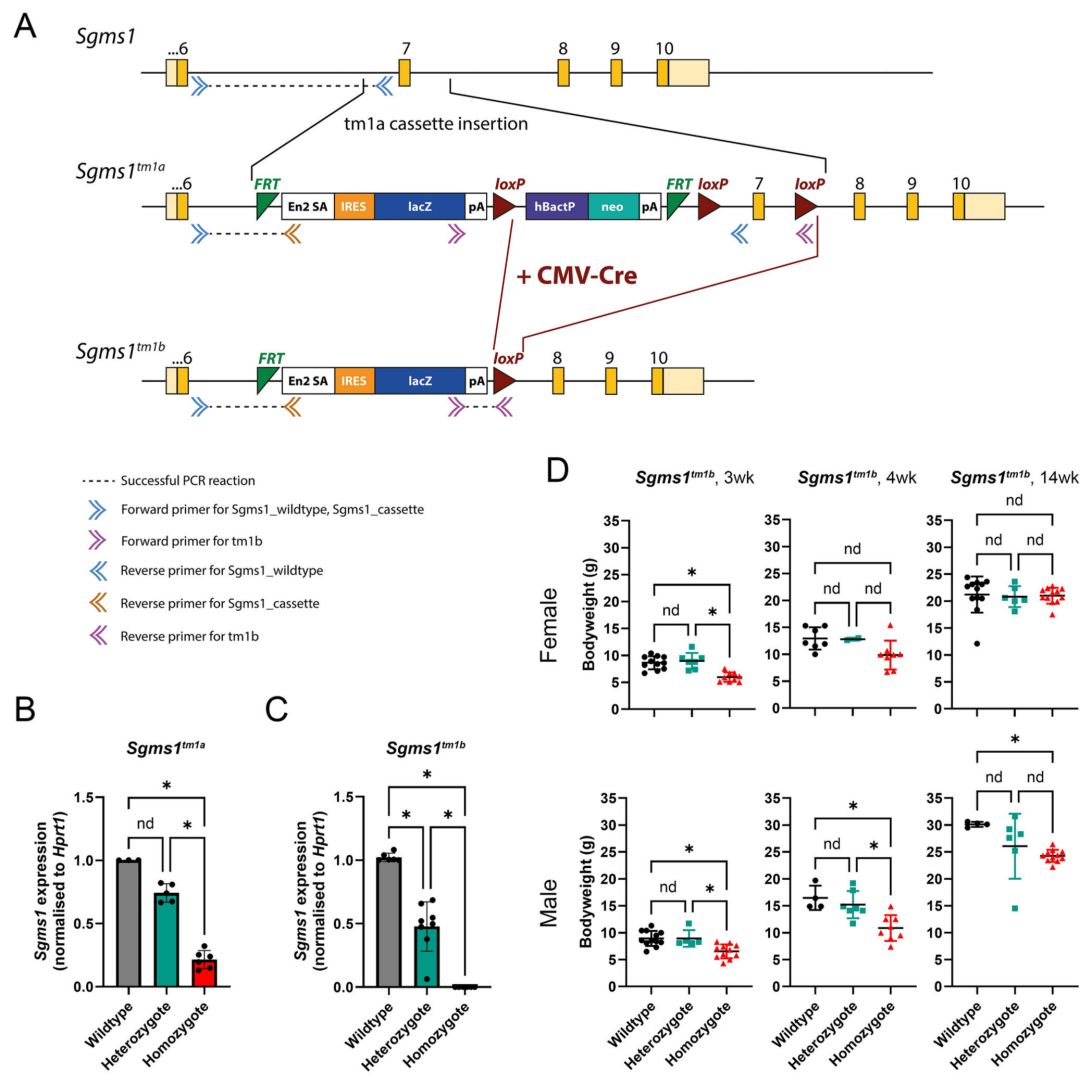
The structure of the *Sgms1* alleles and their impact. **A**, Schematic of the *Sgms1, Sgms1*^*tm1a*^ and *Sgms1*^*tm1b*^ alleles, with exons in yellow and features of the cassette inserted into intron 6–7, including a splice acceptor site (En2 SA), an internal ribosome entry site (IRES) and a β-galactosidase reporter (lacZ), followed by a neomycin resistance marker (neo) expressed from an independent β-actin promoter (hBactP). FRT sites (green) surround the inserted cassette and LoxP sites (dark red triangles) flank exon 7 (https://www.mousephenotype.org/data/genes/MGI:2444110; Skarnes et al., 2011). The lacZ-tagged *Sgms1*^*tm1b*^ allele was generated by breeding *Sgms1*^*tm1a*^-carrying mice to mice expressing Cre recombinase driven by the CMV promoter, to delete the floxed exon 7 and the neomycin-containing promoter-driven selection cassette. Arrows indicate sites of primer binding. Not to scale. **B, C**, RT-qPCR expression levels of *Sgms1* mRNA relative to the housekeeping gene *Hprt* in inner ears from *Sgms1*^*tm1a*^ (**B**) and *Sgms1*^*tm1b*^ (**C**) homozygotes (red), heterozygotes (teal) and littermate wildtypes (grey). Means ± SD normalised to the WT levels are plotted, with black symbols showing individual mouse values. There is about 22% of residual transcript of *Sgms1* in inner ears of *Sgms1*^*tm1a/tm1a*^ mice, but no *Sgms1* transcript was detected in inner ears of *Sgms1*^*tm1b/tm1b*^ mice. *Sgms1*^*tm1a*^ *n* = 3 wildtypes, 5 heterozygotes, 6 homozygotes. *Sgms1*^*tm1b*^ *n* = 6 wildtypes, 8 heterozygotes, 6 homozygotes. * - indicates FDR-adjusted *p* < 0.05. nd – no significant discovery (Kruskal-Wallis one-way ANOVA followed by linear step-up FDR correction). **D**, Weights of *Sgms1*^*tm1b*^ mice plotted with age, showing the reduced size of male homozygotes (red) compared with their control littermates at all ages (wildtype, black and heterozygotes, teal), while female homozygotes (red) show reduced weights only at 3 weeks old. Three weeks old: *n* = 11 wildtype females, 7 heterozygote females, 9 homozygote females, 12 wildtype males, 5 heterozygote males, 11 homozygote males. Four weeks old: *n* = 7 wildtype females, 2 heterozygote females, 8 homozygote females, 4 wildtype males, 7 heterozygote males, 8 homozygote males. Fourteen weeks: *n* = 12 wildtype females, 6 heterozygote females, 12 homozygote females, 4 wildtype males, 6 heterozygote males, 11 homozygote males. * - indicates FDR-adjusted *p* < 0.05. nd – no significant discovery (Kruskal-Wallis one-way ANOVA followed by linear step-up FDR correction).

The *Sgms1*^*tm1a*^ mutant mice were obtained from the Wellcome Sanger Institute Mouse Genetics Project (White et al., 2013). The mutant allele carries a large promoter-driven cassette designed to interrupt normal gene transcription and exon 7 (ENSMUSE00001279700, transcript ENSMUST00000142618) is surrounded by LoxP sites (Fig 1A; ES cell EPD0725_2_G05; Skarnes et al., 2011). The allele is designated *Sgms1*^*tm1a(EUCOMM)Wtsi*^, abbreviated to *Sgms1*^*tm1a*^ in this report. The colony was maintained on a genetic background of C57BL/6 N; C57BL/6N-*A*^*tm1Brd*^*/a*. To generate the *Sgms1*^*tm1b*^ allele, *Sgms1*^*tm1a*^ mice were crossed to *Hprt*^*Tg(CMV-Cre)Brd/Wtsi*^ transgenic mice on a C57BL/6 N genetic background with systemic expression of Cre recombinase to recombine between LoxP sites and remove exon 7 of *Sgms1* (Fig. 1A). Mice showing the correct excision were mated to wildtype C57BL/6 N mice. The Cre recombinase allele was removed by selective breeding and the subsequent *Sgms1*^*tm1b*^ colony was maintained by intercrossing. Mice were genotyped using PCR using primers listed in Table 1. Mutant mice are available through the European Mouse Mutant Archive.

### 2.3 Real-time quantitative PCR

Expression levels of both *Sgms1*^*tm1a*^ and *Sgms1*^*tm1b*^ mutants were measured. The inner ear of postnatal day (P)4 homozygous, heterozygous and wildtype littermate mice was dissected in RNAlater. Total RNA was extracted with QIAshredder columns (QIAgen, cat. no. 79654) and the RNAeasy mini kit (QIAgen, cat. no. 74104). RNA was normalized to the same concentration for cDNA synthesis using oligo dT and Super-Script II (Invitrogen). Real-time PCR was performed in triplicate technical repeats for each sample on a CFX Connect real time PCR machine (BIO-RAD). The *Sgms1* probe used was chosen from the ABI bank with optimal cover of the 3′ untranslated region (Applied Biosystem, Mm00522643_m1). The housekeeping gene Hypoxanthine-guanine phospharibosyltransferase (*Hprt*) was amplified simultaneously (Applied Biosystem, Mm01318747_g1) as an internal reference. The relative quantity of *Sgms1* was calculated using the 2^-ΔΔCt^ method (Livak and Schmittgen 2001). The Kruskal-Wallis test was used to carry out a nonparametric one-way ANOVA comparing all three genotypes, followed by a two-stage linear step-up procedure (Benjamini et al., 2006) to carry out pairwise group comparisons and control for the false discovery rate. Statistical analysis was carried out using GraphPad Prism v10.2 (GraphPad Software, Boston, USA).

### 2.4 Reporter assay for expression of Sgms1 in inner ear

X-gal staining was employed to reveal the expression pattern of *Sgms1* in the inner ear using the *LacZ* gene in the cassette of the *Sgms1*^*tm1b*^ allele as a reporter (Fig. 1A). Inner ears of P21 *Sgms1*^*tm1b*^ heterozygotes (*n* = 3) and a littermate wildtype negative control (*n* = 1) were fixed in 4% paraformaldehyde (PFA) for 2 hrs at room temperature, followed by PBS washes and decalcification in 10% EDTA until soft. Samples were treated with Solution A (2 mM MgCl_2_; 0.02% NP-40; 0.01% sodium deoxycholate; PBS) for 30 mins, then incubated with Solution B (Solution A plus 5 mM K_3_Fe(CN)_6_; 5 mM K_4_Fe(CN)_6_; 1 mg/ml X-gal in DMSO) overnight at 37 ^◦^C. After PBS washes, samples were immersed in 30% sucrose, then embedded in OCT compound. Cryosections were cut at 14–16 μm thickness. Sections were rinsed in water, dehydrated and cleared, mounted and examined by bright-field microscopy.

### 2.5 Immunohistochemistry

Paraformaldehyde-fixed, paraffin-embedded inner ear samples of wild type C57BL/6 N mice aged P4 (*n* = 3) and P14 (*n* = 7) were sectioned at 8 µm and slides were stained using the Ventana Discovery machine and reagents according to the manufacturer’s instructions (DABMap™ Kit (cat.no 760–124), Hematoxylin (cat.no 760–2021), CC1 (cat.no 950–124), EZPrep (cat.no 950–100), LCS (cat.no 650–010), RiboWash (cat.no 760–105), Reaction Buffer (cat.no 95–300), and RiboCC (cat.no 760–107)). The primary antibody used was rabbit anti-SGMS1 antibody (1:800; Atlas Antibodies, HPA045191). diluted in staining solution (10% foetal calf serum, 0.1% Triton, 2% BSA and 0.5% sodium azide in PBS), the secondary antibody was anti-rabbit IgG (Jackson ImmunoResearch, cat.no 711–065–152, diluted 1:100) and labelling was visualised using the 3,3′-diaminobenzidine (DAB) method with a blue counterstain (Bluing reagent (cat.no 760–2037)). A Zeiss Axioskop 2 microscope was used to examine slides, and photos were taken using a Zeiss Axiocam camera and the associated Axiocam software. The primary antibody was omitted in some sections as a negative control.

### 2.6 Auditory brainstem responses (ABR)

Mice were anaesthetised using ketamine hydrochloride (100 mg/Kg, Ketaset®, Fort Dodge Animal Health) and xylazine hydrochloride (10 mg/Kg, Rompun®, Bayer Animal Health) and subcutaneous needle electrodes were inserted on the vertex (active), and over the left (reference) and right (ground) bullae. A calibrated sound system was used to deliver free-field click (0.01 ms duration) and tone pip (various frequencies from 3 to 42 kHz, 5 ms duration, 1 ms rise/fall time) stimuli at a range of intensity levels in 5 dB steps. Averaged responses to 256 stimuli, presented at 42.2 per second, were analysed and thresholds established as the lowest sound intensity giving a visually-detectable ABR response (any wave). Threshold estimates were found to be normally-distributed (Shapiro-Wilk test, *p* > 0.05). Two-Way ANOVA was used to compare across genotypes at each age, with Tukey’s multiple comparisons tests used to identify which stimuli had a significant threshold elevation.

### 2.7 Distortion product otoacoustic emission (DPOAE) measurements

DPOAEs were recorded from 14-week old *Sgms1*^*tm1b*^ homozygotes (*n* = 5), heterozygote (*n* = 1) and wildtype littermates (*n* = 4). The DPOAE is a sound generated by the cochlea following the presentation of two simultaneous long-lasting pure tones (f_1_ and f_2_) at a different frequency in comparison with the stimulus tones (Kemp, 1978). The two distinct tones were presented at a specific frequency ratio, f_2_/f_1_= 1.20. The value of the 2f_1_-f_2_ DPOAE component was extracted from a fast Fourier transform of the recorded microphone signal and plotted as a function of f_2_ level. Mice were anaesthetised with urethane (0.01 ml/g of a 20% solution, IP), a speculum was inserted into the ear canal and the detection probe microphone and sound delivery speakers were sealed into the speculum as described previously (Ingham et al., 2021). The f_2_ tones were presented at 6, 12, 18, 24 and 30 kHz at increasing level 0–65 dB SPL in 5 dB steps, while the f_1_ was presented at 10 dB above the f_2_ (10–75 dB SPL). For each stimulus the DPOAE threshold was determined as the lowest stimulus level (dB SPL) that rose above two standard deviations from the mean noise floor. DPOAE threshold estimates were found to be normally-distributed (Shapiro-Wilk test, *p* > 0.05). Two-Way ANOVA was used to compare across genotypes at each age, with Tukey’s multiple comparisons tests used to identify which stimuli had a significant threshold elevation.

### 2.8 Endocochlear potential measurement

Mice from the *Sgms1*^*tm1b*^ colony were anaesthetized with 0.01 ml/g body weight of 20% urethane, a tracheal cannula was inserted and the bulla was opened to reveal the cochlea while the body temperature was kept at 37 ^◦^C by a feedback-controlled heating pad. A small hole was made in the bony wall of the cochlea over the basal turn of scala media, and a micropipette electrode filled with 150 mM potassium chloride was advanced through the hole and through the lateral wall into the scala media. The potential difference between the scala media and a reference silver/silver chloride pellet under the dorsal skin was recorded as described previously (Steel and Barkway 1989). Measurements of endocochlear potential were not normally-distributed for all genotypes and ages (Shapiro-Wilk test, *p* < 0.05), and therefore were compared using a non-parametric Kruskall-Wallis ANOVA, with Dunn’s multiple comparisons test.

### 2.9 Immunolabelling whole mounts of the cochlear lateral wall

Phalloidin was used to stain filamentous actin, present in the tight junctions between the marginal cells which enabled the identification of the marginal cell boundaries. A goat anti-Kcnq1 antibody was used together with an anti-goat antibody linked to Alexa Fluor 488 to identify this potassium channel present in the apical membrane of marginal cells. 4′,6-diamidino-2-phenylindole (DAPI) was used for the visualisation of DNA in nuclei. Inner ears from the *Sgms1*^*tm1b*^ colony were fixed in 4% PFA at room temperature (RT) for 1–2 h and the cochlear lateral walls were dissected out under PBS. Samples were blocked with 1% BSA in PBS containing 0.1% Triton x 100 for 30 min, then incubated either with goat anti-Kcnq1 polyclonal antibody (1:100 in blocking solution; Santa Cruz, CA, sc-10646) for 4 h at RT or mouse monoclonal anti-Kcnq1 antibody (1:300 overnight at 4 ^◦^C. Samples were washed followed by labelling with Alexa Fluor 594 Phalloidin (1:300 in blocking solution; Invitrogen) and either donkey anti-goat secondary IgG Alexa Fluor 488 or goat anti- mouse secondary IgG1 Alexa Fluor 568 (1:300 in blocking solution; Invitrogen) for 2 h at RT or with Alexa Fluor phalloidin (1:500, 568 or 488). After washing with PBS, samples were mounted in Vectashield Anti-fade Mounting Medium with DAPI (Vector Laboratories; Burlingame, CA) or ProLong gold antifade mountant. Samples were visualised using the LSM 710 confocal microscope (Zeiss, Germany) using the imaging software ZEN 010. The whole length of the stria vascularis was surveyed prior to taking confocal images using the x40 objective with oil. Samples from several ages were analysed: at 3 weeks WT=5, het=2, hom=6; at 4–5 weeks WT=2, het=4, hom=6; at 8–9 weeks WT *n* = 4, het *n* = 7, hom *n* = 1; at 12–17 weeks WT *n* = 4, het *n* = 1, hom *n =* 4; and at 6–7 months WT *n =* 3, het *n =* 4, hom *n =* 3. Colours were altered using FiJi after acquisition for consistency and better visual clarity and to normalise the dynamic range across figure panels.

### 2.10 Immunostaining cryosections of the cochlear lateral wall

Inner ears of 14-week old mice from the *Sgms1*^*tm1b*^ colony were fixed in 4% PFA at RT for 2 h, followed by PBS washes and decalcification in 10% EDTA until soft. After PBS washes, samples were immersed in 30% sucrose, then embedded in OCT compound. Cryosections were cut at 14–16 μm thickness, blocked by incubation with 10% donkey serum (with 0.1% TritonX-100 in PBS) for 1 hour then incubated with primary antibodies in blocking solution overnight at 4 ^◦^C. The antibody used to label sections was rabbit anti-Kcnj10 polyclonal antibody (Alomone labs, 1:400). Acetylated α-tubulin antibody (mouse monoclonal) was also used to label root cells (Invitrogen 32–2500; 1:1000). After labelling, sections were washed with PBS and incubated with the corresponding secondary antibodies at room temperature for 2 h (goat anti-rabbit for Kcnj10, donkey anti-mouse for α-tubulin, Invitrogen, 1:500). After washing with PBS, slides were mounted and imaged by confocal microscopy using a LSM 710 confocal microscope (Zeiss, Germany) and the imaging software ZEN 2010. For Kcnj10 labelling, the number of mice used were WT *n =* 4, *Sgms1*^+*/tm1b*^ heterozygote *n =* 1, *Sgms1*^*tm1b/ tm1b*^ homozygotes *n =* 4. Colours were altered using FiJi after acquisition for consistency and better visual clarity and to normalise the dynamic range across figure panels.

### 2.11 Scanning electron microscopy

The organ of Corti of mice from the *Sgms1*^*tm1b*^ colony aged P28 were examined by scanning electron microscopy to assess the condition of the hair cell surfaces. Cochleas of WT (*n* = 2), heterozygous (*n* = 1) and homozygous mutants (*n* = 3) were fixed for two hours in 2.5% glutaraldehyde in 0.1 M sodium cacodylate buffer with 2 mM CaCl_2_. Samples were then fine-dissected in PBS to expose the organ of Corti and processed according to the osmium tetroxide-thiocarbohydrazide (OTOTO) method (Hunter-Duvar 1978) before dehydration through an ethanol series, critical point drying, and mounting. Regions of the cochlea were identified using the frequency-place map (Müller et al., 2005). Images were taken using a JEOL JSM 7800 Prime scanning electron microscope. A standard magnification of 60x was used to view the whole length of the organ of Corti, and higher magnifications were used for close-ups on hair cell rows (2000x) and individual hair cells (15000–23000x). Whole images were adjusted using Adobe Photoshop to normalise the dynamic range across all panels.

### 2.12 Gene expression analysis using the gEAR

Gene expression in the mouse inner ear was assessed using single cell RNAseq data obtained from the gEAR portal (https://umgear.org, accessed December 2021; Orvis et al., 2021). We chose datasets to include multiple ages and cell types (embryonic day (E)16, postnatal day (P)1, P7 (Kolla et al., 2020), P15 (Ranum et al., 2019), P20 (Xue et al., 2021), P30 (Korrapati et al., 2019), and spiral ganglion neuron datasets at young adult stages (Shrestha et al., 2018; Petitpre et al., 2018), and normalised expression within each dataset and cell type to *Hprt* expression. Cell types were defined by the authors of the original experiment. Where there was more than one set of measurements for a cell type and age (eg the E16 dataset has “OHC_1″ and “OHC_2″, both representing outer hair cells), normalised expression levels were averaged. Fifteen marker genes were chosen for comparison (*Myo7a* for hair cells, *Fgf8* for inner hair cells, *Slc26a5* for outer hair cells, *Sox2* for non-sensory cells, *S100b* for inner pillar cells, *Hes5* for Deiters’ cells, *Prss36* for Claudius cells, *Epha5* for Reissner’s membrane, *Kcne1* for marginal cells, *Met*-for intermediate cells, *Cldn11* for basal cells, *Slc26a4* for spindle and root cells, *Ifitm1* for fibrocytes, *Slc17a7* for Type 1 spiral ganglion neurons and *L1cam* for type 2 spiral ganglion neurons). These are either well-established marker genes for their cell types (*Myo7a, Fgf8, Slc26a5, Sox2, S100b, Hes5, Kcne1, Met, Cldn11, Slc26a4*) or were chosen because they exhibited specific strong expression in the gEAR datasets for the cell type in question (*Prss36, Epha5, Ifitm1, Slc17a7, L1cam*).

### 2.13 Human association analysis

The 1958 British Birth Cohort and the collection of hearing data and analysis have been described previously (Strachan et al., 2007; Ecob et al., 2008; Nolan et al., 2013). Participants were drawn from 17,638 individuals born in England, Scotland, and Wales in one week of March 1958. Of the original cohort, 9377 members were revisited by a research nurse for a biomedical follow-up in 2002–2004. Hearing measures consisted of pure tone audiometry at 1 kHz and 4 kHz at age 44–45 years and were adjusted for sex, nuisance variables (noise at test, nurse performing test, audiometer used in test), conductive loss, and hearing loss in childhood. DNA was collected from 6099 individuals and genotyped on various Illumina and Affymetrix SNP chips (for detail, see https://www.metadac.ac.uk/1958-birth-cohort/genetic-resource/). These data were then imputed to the 1000 Genomes haplotypes (released March 2012) using MACH and Minimac. Measured SNPs with >95% call rate and Hardy–Weinberg p-value >0.0001 were included as the input set. In subsequent analysis, imputed SNPs with low imputation quality (r2-hat < 0.3 or MAF < 1%) were omitted. Individual associations were performed to hearing thresholds at 1 kHz and 4 kHz.

## 3 Results

### 3.1 Incomplete knockdown of Sgms1 transcription from the Sgms1^tm1a^ allele

The *Sgms1*^*tm1a*^ mutation was designed as a knockout-first, conditional-ready allele in which a large DNA cassette was inserted in the intron between exons 6 and 7 in order to disrupt transcription of the gene (Fig. 1A; Skarnes et al., 2011). These mutant alleles can sometimes be leaky (White et al., 2013), so we assessed the degree of knockdown using qRT-PCR on whole inner ear tissue from homozygotes, heterozygotes and wildtype littermate mice at 4 days old. In the homozygous mutants, expression levels were around 20% of normal wildtype levels, while heterozygotes showed an intermediate level (Fig 1B). As *Sgms1*^*tm1a*^ homozygotes showed normal ABR thresholds (Ingham et al., 2019), we concluded that 20% of the normal amount of transcription was enough to support normal hearing.

### 3.2 Generating a null allele of Sgms1

To generate a more severe mutation, we crossed the *Sgms1*^*tm1a*^ mutants to a line carrying a CMV-driven Cre recombinase gene on the same pure C57BL/6 N genetic background as the original mutation. Cre recombinase recognises LoxP sites in the inserted cassette (red triangles in Fig 1A) and deletes the DNA between them, which in this case includes exon 7 of the *Sgms1* gene. The resulting allele, *Sgms1*^*tm1b*^, is predicted to have a frameshift as exon 7 has a number of bases that is not divisible by 3, so if any mRNA is produced it would be expected to encode an abnormal amino acid sequence following exon 6, a premature stop codon, and nonsense-mediated decay of the mRNA. The effect of this new allele was assessed using qRT-PCR on 4-day old inner ear samples and no transcript was detectable in homozygotes (Fig. 1C). Transcription was reduced to around half of normal levels in the heterozygotes.

In addition to male infertility shown by both mutant alleles, the new *Sgms1*^*tm1b*^ homozygotes showed reduced viability, with only 111 homozygotes out of 1141 offspring from heterozygote intercrosses, 9.7% compared with the expected 25%. For the *Sgms1*^*tm1a*^ homozygotes, 22 out of 99 total offspring were produced from heterozygous intercrosses, a rate close to Mendelian expectations. Male *Sgms1*^*tm1b*^ homozygotes were smaller than their wildtype littermates from 3 weeks to 14 weeks old and female homozygotes were also smaller at 3 weeks old but their weights later caught up with the weights of their control littermates (Fig. 1D).

### 3.3 Expression of Sgms1 in the cochlea

**Fig. 2:**
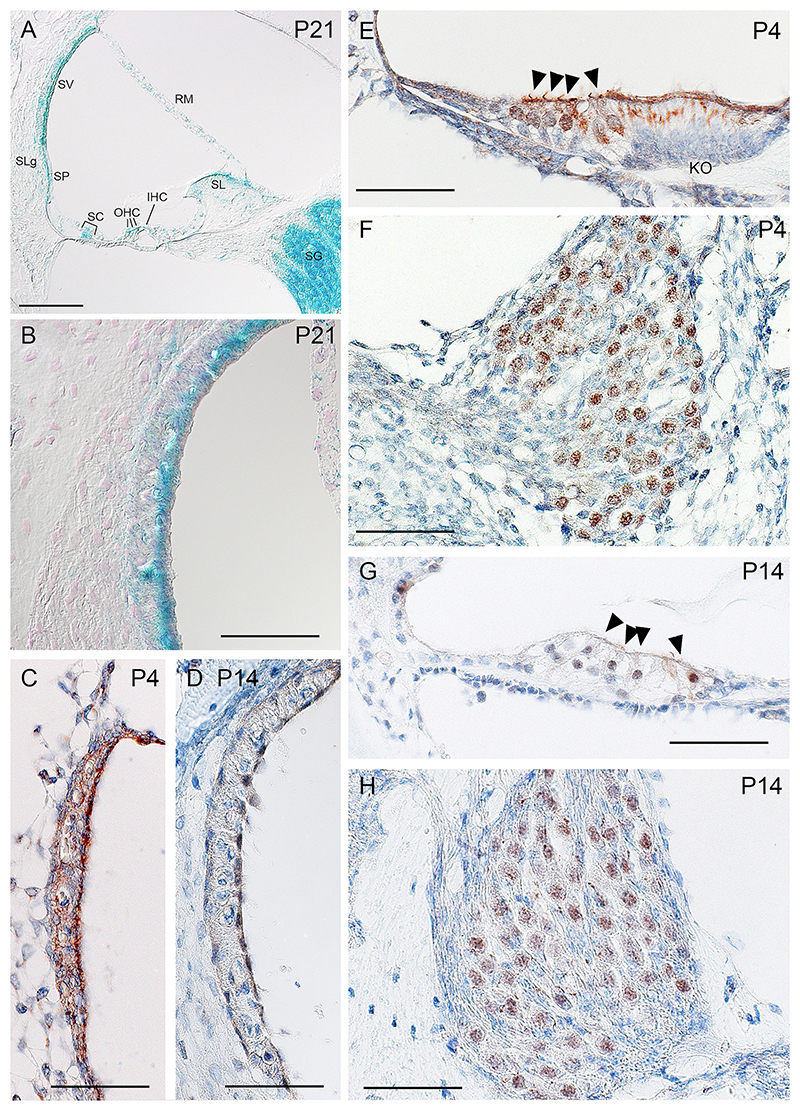
Expression of the *Sgms1* gene in the cochlear duct. **A, B**, X-gal staining (blue) of the P21 *Sgms1*^+*/tm1b*^ cochlea (*n* = 3) indicated that *Sgms1* is strongly expressed in the spiral ganglion (SG) and the luminal side of the stria vascularis (SV) as well as in the organ of Corti, spiral limbus (SL), spiral prominence (SP), parts of the spiral ligament (SLg) and Reissner’s membrane (RM). SC: supporting cells; OHC: outer hair cells; IHC: inner hair cells. Scale bar = 100 µm. The *Sgms1*^+*/*+^ cochlea showed no labelling (*n* = 1; not shown). **B**, Higher magnification of the stria vascularis showing strong labelling of marginal cells and also intermediate and basal cell labelling. Scale bar = 50 µm. **C**–**H**, Immunohistochemistry using an antibody to Sgms1 showed strong staining (brown) in the spiral ganglion (**F, H**), the stria vascularis (**C, D**), outer and inner hair cells (**E, G**, arrowheads). At P4, staining was also seen in Kölliker’s organ (KO) (**E**). C57BL/6 N wildtype mice aged P14 (**D, G, H**) (*n* = 7) and P4 (**C, E, F**) (*n* = 3). Scale bar 50 µm.

The presence of a reporter gene, *LacZ* encoding β-galactosidase, in the inserted DNA in the mutant allele enabled the visualisation of cells within the cochlea that would normally express the *Sgms1* gene. Expression is shown by blue staining in sections of the cochlea of *Sgms1*^+*/tm1b*^ heterozygotes (Fig 2A, B). The spiral ganglion and marginal cells of the stria vascularis both showed strong and consistent labelling at the age studied (P21), but there was widespread staining of other cells around the cochlear duct including outer hair cells, supporting cells (Claudius cells, inner phalangeal cells, pillar cells and Hensen’s cells), spiral limbus cells, spiral prominence cells, root cells, basal and intermediate cells of the stria and Reissner’s membrane.

Immunohistochemistry was carried out at younger stages, P4 and P14, in wildtype C57BL/6 N mice using an antibody to human SGMS1. Both stages showed expression in the cochlear duct, especially in the stria vascularis, hair cells and supporting cells of the organ of Corti, and the spiral ganglion (Fig 2C–H). At P4, staining was also observed in Kölliker’s organ. No brown labelling was detected when the primary antibody was omitted (Suppl Fig 1).

An analysis of published single cell sequence data deposited in the gEAR database suggested wide expression of *Sgms1* in multiple cell types of the cochlear duct, and a similar pattern of expression changes with age for *Sgms1* as for known hair cell markers like *Myo7a* (all hair cells) and *Slc26a5* (outer hair cells) (Suppl Fig. 2).

### 3.4 Sgms1^tm1b^ homozygotes show slowly progressive hearing loss

**Fig. 3:**
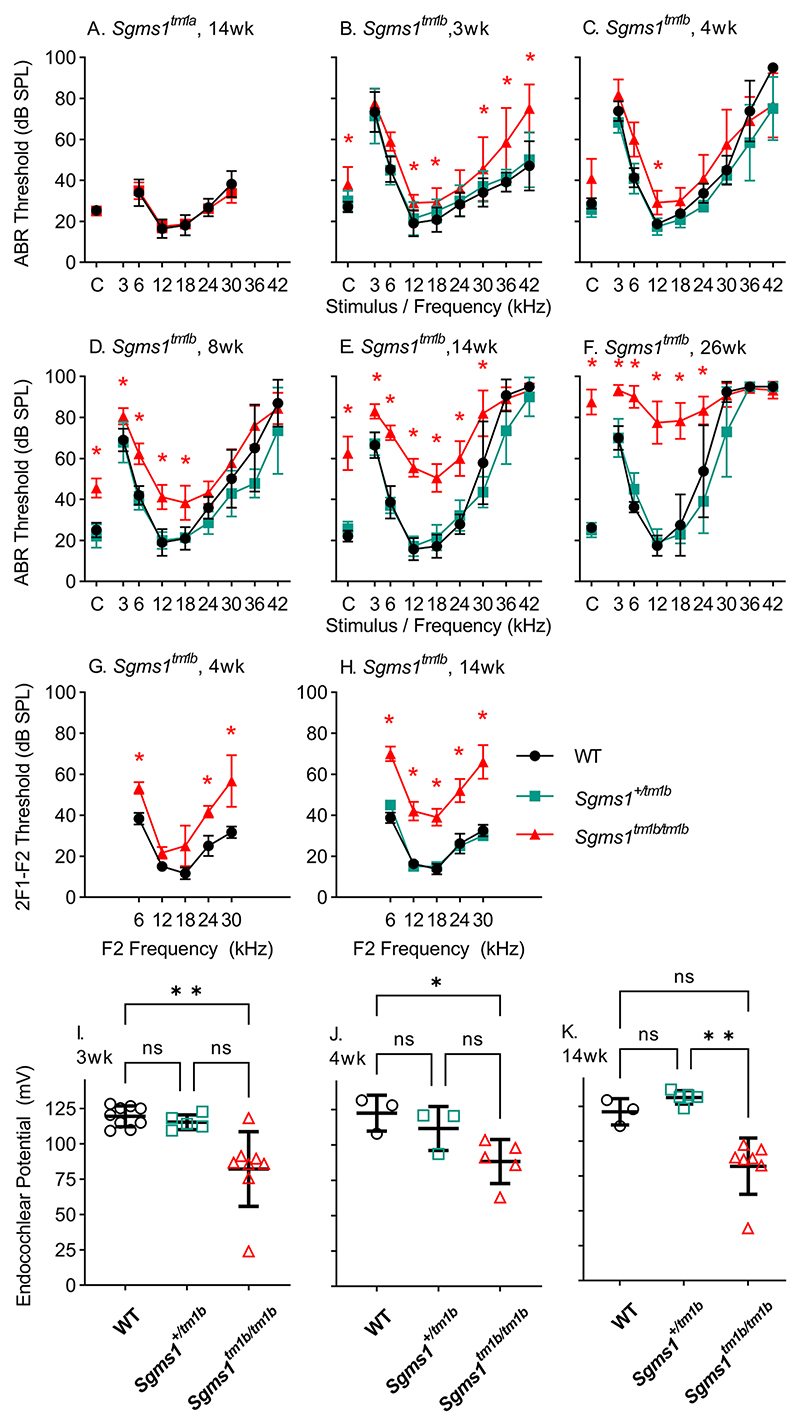
Auditory responses in *Sgms1*^*tm1a*^ and *Sgms1*^*tm1b*^ alleles. **A**, ABR thresholds (mean ± SD) from *Sgms1*^*tm1a/tm1a*^ (magenta, *n* = 4) and wildtype mice (black, *n* = 14) from the same genetic background at 14 weeks old. Data replotted from Ingham et al. (2019). Thresholds for these groups of mice did not differ significantly (ANOVA, F (1, 96) = 0.1641, *p =* 0.6863). **B-F**, ABR thresholds (mean ± SD) from *Sgms1*^*tm1b/tm1b*^ (magenta), *Sgms1*^+*/tm1b*^ (teal) and littermate *Sgms1*^+*/*+^ (black) mice at ages from 3 weeks to 6 months old. The number of *Sgms1*^*tm1b/tm1b*^, *Sgms1*^+*/tm1b*^ and *Sgms1*^+*/*+^ mice tested, respectively, are: 3 weeks 10, 7, 12; 4 weeks 6, 6, 4; 8 weeks 9, 7, 5; 14 weeks 10, 7, 7; 6 months 6, 5, 4. A subset of the mice from 4 weeks onwards were tested at repeated ages. The progressive increase in thresholds at high frequencies in all genotypes are associated with the C57BL/6 N genetic background. Significant threshold elevations in *Sgms1*^*tm1b/tm1b*^ mutants were noted at 3 weeks (ANOVA, F (2, 234) = 56.20, *p* < 0.0001), 4 weeks (ANOVA, F (2, 117) = 22.66, *p* < 0.0001), 8 weeks (ANOVA F (2, 162) = 87.26, *P* < 0.0001), 14 weeks (ANOVA, F (2, 189) = 288.9, *p* < 0.0001) and 26 weeks (ANOVA, F (2, 108) = 228.0, *p* < 0.0001). Results of Tukey’s multiple comparisons tests are indicated on each panel, where significant elevations of threshold between *Sgms1*^*tm1b/tm1b*^ and *Sgms1*^+*/*+^ are indicated by red asterisks (*p* < 0.05). **G**,**H** DPOAE thresholds (mean ± SD) plotted as a function of f2 frequency at 4 and 14 weeks old from *Sgms1*^*tm1b/tm1b*^ (magenta), *Sgms1*^+*/tm1b*^ (teal) and littermate *Sgms1*^+*/*+^ (black) mice. The number of *Sgms1*^*tm1b/tm1b*^, *Sgms1*^+*/tm1b*^ and *Sgms1*^+*/*+^ mice tested, respectively, are: 4 weeks 3, 0, 4; 14 weeks 5, 1, 4. Significant threshold elevations were noted in *Sgms1*^*tm1b/tm1b*^ mutants at 4 weeks (ANOVA, F (1, 20) = 52.90, *p* < 0.0001) and 14 weeks (ANOVA, F (2, 35) = 232.0, *p* < 0.0001). Results of Tukey’s multiple comparisons tests are indicated on each panel, where significant elevations of threshold between *Sgms1*^*tm1b/tm1b*^ and *Sgms1*^+*/*+^ are indicated by red asterisks (*p* < 0.05). **I-K**, Endocochlear potentials recorded from *Sgms1*^*tm1b*^ mice at (**I**) 3, (**J**) 4 and (**K**) 14–15 weeks old. Open symbols show individual recordings and the mean ± SD is plotted. The number of *Sgms1*^*tm1b/tm1b*^, *Sgms1*^+*/tm1b*^ and *Sgms1*^+*/*+^ mice tested, respectively, are: 3 weeks 8, 5; 9; 4 weeks 5, 3, 3; 14–15 weeks 7, 5, 3. Results of Kruskall-Wallis ANOVA across age indicated significant differences in EP magnitude between genotypes; 3 weeks, KW-statistic = 11.78, *p* = 0.0006; 4 weeks, KW-statistic = 6.376, *p* = 0.0203; 14–15 weeks, KW-statistic = 11.63, *p* < 0.0001. Dunn’s multiple comparisons test results are indicated by ns (not significant), * *p* < 0.05, ** *p* < 0.01.

ABR recordings revealed a gradual increase in thresholds in *Sgms1*^*tm1b/tm1b*^ homozygotes with increasing age from 3 weeks to 6 months old (Fig. 3B-F). Heterozygote thresholds were similar to those of the wildtype littermates. All three genotypes showed raised thresholds at the highest frequencies tested due to the known age-related hearing loss associated with the C57BL/6 N genetic background (Fig. 3B–F). ABR thresholds of *Sgms1*^*tm1a/tm1a*^ mutants are replotted from Ingham et al. (2019) for comparison (Fig. 3A). Distortion product otoacoustic emissions (DPOAEs) thresholds at 4 and 14 weeks old were also raised (Fig. 3G,H) indicating that outer hair cell function was affected by the mutation.

Endocochlear potentials (EP) were recorded at 3, 4 and 14 weeks old. Heterozygotes had similar EP levels compared with wildtypes, but homozygous mutants had EPs that were reduced by between 30 and 40 mV on average compared with their wildtype littermates at each age studied (Fig 3I-K). EP did not become any smaller with increasing age in the homozygous mutants (Suppl fig 3)

### 3.5 Hair cells appear to develop normally

**Fig. 4:**
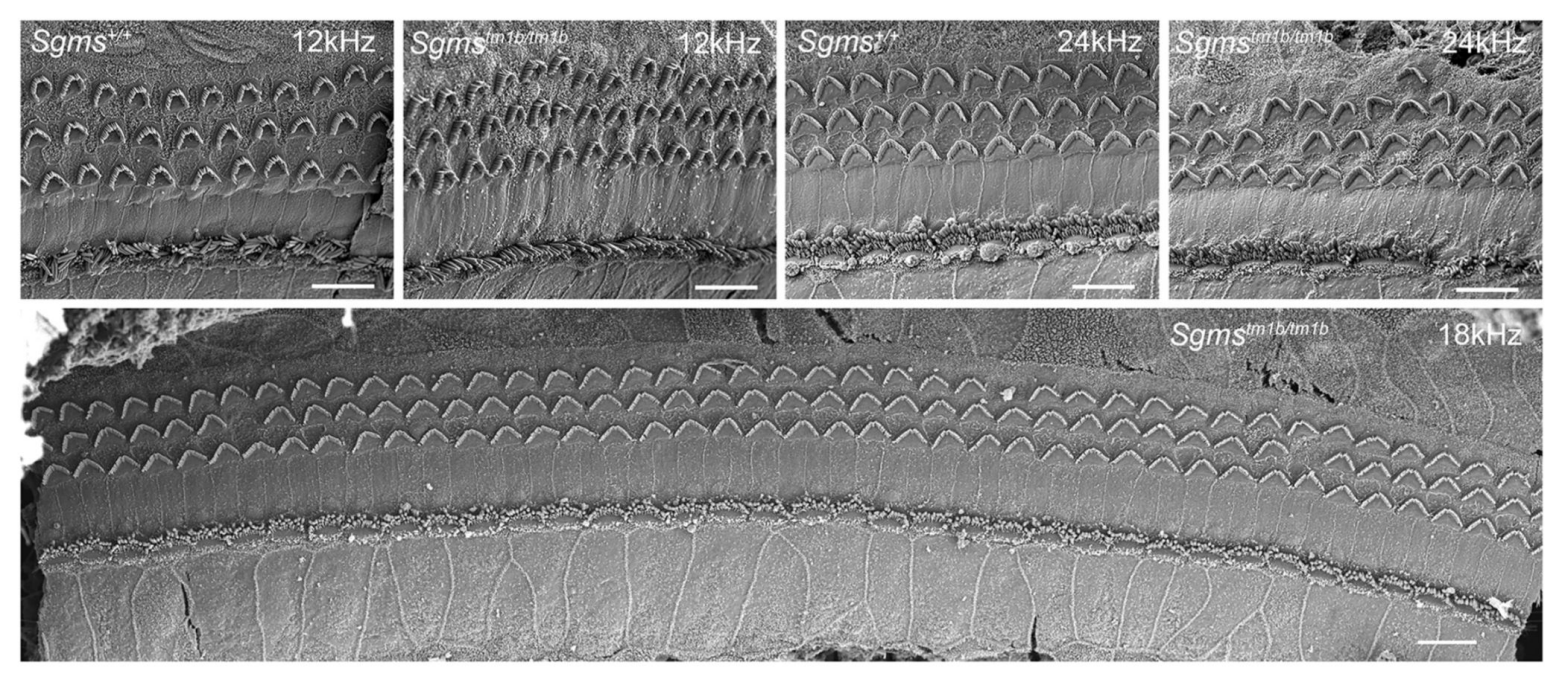
Scanning electron microscopy of *Sgms1*^*tm1b/tm1b*^ mutants. Scanning electron microscopy of *Sgms1*^*tm1b/tm1b*^ and wildtype mice aged 4 weeks at the indicated best-frequency regions of the cochlea, showing a normal arrangement of hair cells and minimal loss of stereocilia bundles in the mutants. Scale bar 10 µm. Numbers of mice examined: 3 *Sgms1*^*tm1b/tm1b*^, 1 *Sgms1*^+*/tm1b*^ and 2 *Sgms1*^+*/*+^.

There are early signs of an increase in ABR thresholds by 4 weeks of age in homozygotes, so this age was selected to examine by scanning electron microscopy to look for any early structural correlates of the dysfunction. The overall organisation of cell types within the organ of Corti was normal in the mutants compared with littermate controls. Stereocilia bundles of mutant hair cells were indistinguishable from those of control mice (Fig. 4). However, sporadic gaps were seen in the regular array of outer hair cells at intervals along the length of the cochlear duct in mutants (Fig. 4).

### 3.6 Early stria vascularis disorganisation

**Fig. 5:**
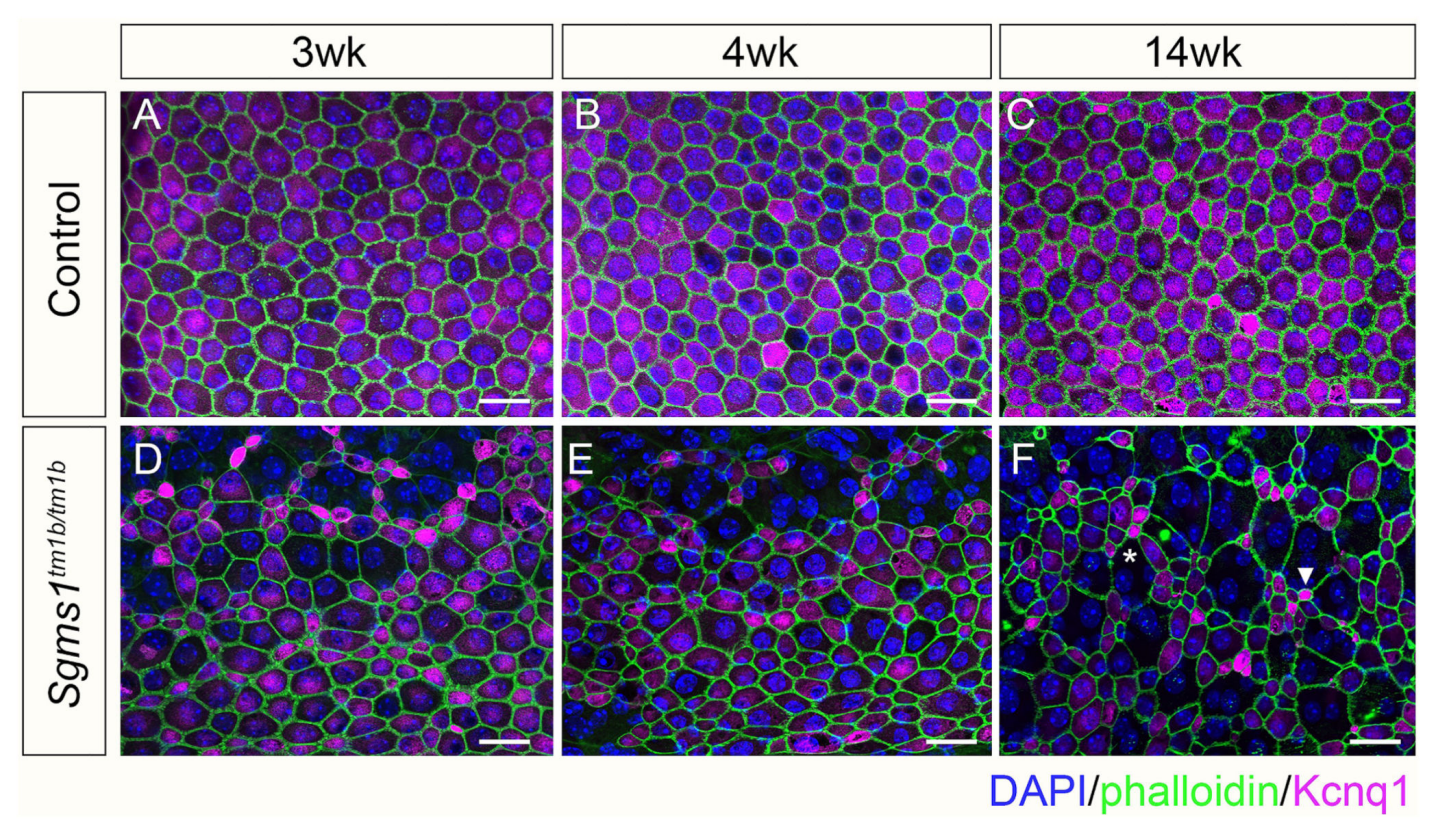
Stria vascularis of *Sgms1*^*tm1b/tm1b*^ mutants. Immunofluorescent labelling of marginal cell boundaries (phalloidin, green) and Kcnq1 expression (magenta) on the apical surface of the stria vascularis in control (wildtype and *Sgms1*^+*/tm1b*^ heterozygotes, **A-C**) and *Sgms1*^*tm1b/tm1b*^ homozygotes (**D-F**) at 3 weeks (**A, D**), 4 weeks (**B, E**) and 14 weeks old (**C, F**). Nuclei are stained by DAPI (blue). Confocal maximum projection images of whole mount preparations. (**A-C)** Marginal cells of control mice have regular hexagonal morphologies of similar sizes and each cell shows positive Kcnq1 labelling at all ages examined. **(D-F)** Marginal cell boundaries of homozygotes show highly variable surface areas including large foci of disorganised boundaries with no detectable Kcnq1 expression (asterisk in **F**) and small surface areas with strong Kcnq1 labelling (arrowhead in **F**). Scale bar 20 µm. Numbers of mice examined: 3 weeks, 6 homozygotes, 2 heterozygotes, 5 wildtypes; 4 weeks, 6 homozygotes, 4 heterozygotes, 2 wildtypes; 14–17 weeks, 4 homozygotes, 4 wildtypes; 6–9 months, 4 homozygotes, 5 heterozygotes, 4 wildtypes.

As the EP was reduced from as early as 3 weeks old in *Sgms1*^*tm1b/tm1b*^ homozygous mutants, we investigated several markers in the lateral wall which are critical for normal EP formation. Firstly, marginal cells of the stria vascularis were examined by confocal microscopy of whole mount preparations. Phalloidin was used to label the boundaries of the marginal cells on the luminal surface of the stria. Control samples showed a regular array of marginal cells with very similar surface areas (Fig 5A–C), with very few instances of small patches of abnormal organisation limited to the extreme of the apical turn. In contrast, the strias of homozygotes had multiple small patches of marked irregularity of marginal cell surfaces giving a disorganised appearance scattered along the cochlear duct from as early as 3 weeks old (Fig 5D-F). The mutant boundaries became more rounded and there were more very large (asterisk in Fig 5F) and very small (arrowhead in Fig 5F) marginal cell surfaces in older samples at 14 weeks and older. As in samples from younger mice, these patches of abnormal organisation were spread along the entire length of the cochlear duct. Enlarged marginal cell surfaces were often accompanied by reduced or absent immunolabelling of Kcnq1, which normally is strongly expressed by marginal cells at their luminal surface (Sakagami et al., 1991; Wangemann 2002; Fig. 5D-F).

**Fig. 6:**
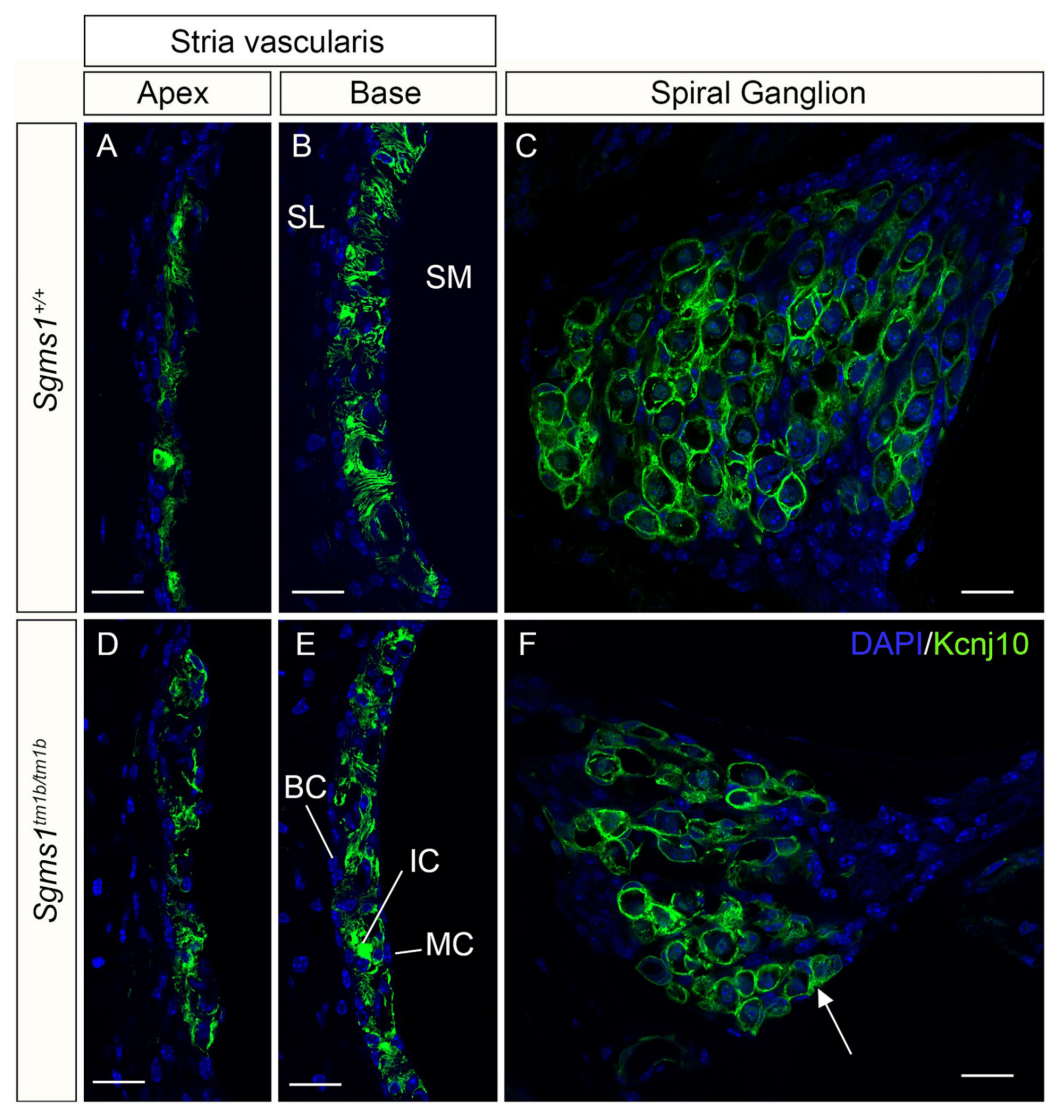
Expression of Kcnj10 in the *Sgms1*^*tm1b/tm1b*^ mutant cochlea. **A-F**, Immunofluorescent labelling of Kcnj10 (green) in sections of the cochlea from mice aged 12–14 weeks. Nuclei are stained by DAPI (blue). The intermediate cells of the stria vascularis are labelled in both homozygotes (**D, E**) and wildtypes (**A, B**), in the apex (**A, D**) and the base (**B, E**). Satellite cells in the spiral ganglion are also labelled in both mutants (**F**) and wildtypes (**C**). Scale bar 20 µm. The numbers of mice used were WT *n* = 4, *Sgms1*^+*/tm1b*^ heterozygotes *n* = 1, *Sgms1*^*tm1b/tm1b*^ homozygotes *n* = 4. SL, spiral ligament; SM, scala media; MC, marginal cells; IC, intermediate cells; BC, basal cells. Arrow in **F** points to satellite cell labelling.

Kcnj10 is expressed in strial intermediate cells and has an important role in normal EP formation (Wangemann et al., 2004; Marcus et al., 2002), so immunolabelling of Kcnj10 was carried out in sections of the cochlea. In contrast to the marginal cell abnormalities seen in whole mounts, the intermediate cells of *Sgms1*^*tm1b/tm1b*^ homozygotes did not show any obvious abnormal labelling at 14 weeks old (Compare Fig. 6A, B with D, E). Kcnj10 is also expressed in the spiral ganglion, and labelling there was similar in homozygous mutants compared with wildtypes (Fig.6C, F).

### 3.7 SGMS1 is associated with human auditory thresholds

No single gene mutations of *SGMS1* have yet been linked to human deafness in families, so we investigated whether it has any role in hearing ability in the general population. A candidate gene association analysis was used, testing genomic markers within 0.1 Mb up- and downstream of the *SGMS1* gene for association with auditory thresholds measured at 44–45 years old in 6099 individuals born during one week in the UK 1958 British Birth Cohort. Genetic data were imputed to the 1000 Genomes dataset (The 1000 Genomes Project Consortium, 2015). We found a significant association of markers close to *SGMS1* with auditory thresholds at both 1 kHz (p value 0.001725; peak marker rs138982939) and 4 kHz (p value 0.000475; peak marker rs139282282), suggesting that this gene may play a role in normal variation of hearing ability in the human population.

## 4 Discussion

We previously found that *Sgms1*^*tm1a*^ homozygotes showed normal ABR thresholds (Ingham et al., 2019), which was unexpected because of the earlier report of a different mouse *Sgms1* mutation causing hearing impairment (*Sgms1*^*tm1Kenw*^ also known as *Sms1*^-^; Lu et al., 2012). The original mutation was on a mixed 129/Sv-C57BL/6 genetic background while the *Sgms1*^*tm1a*^ mutation that we studied was on a C57BL/6 N genetic background, which might have led to a different phenotype.

However, in the current study the *Sgms1*^*tm1a*^ allele was found to be leaky, allowing 20% of the normal level of transcript to be produced. This degree of knockdown still affects other aspects of the phenotype, such as male fertility, but clearly 20% of transcript was enough to support normal hearing.

The *Sgms1*^*tm1b*^ allele that we produced resulted in no detectable transcript in the inner ears of homozygotes and around half the transcript level in heterozygotes. Heterozygotes had normal ABR thresholds, confirming that reduced levels of *Sgms1* transcript are sufficient for normal auditory function. *Sgms1*^*tm1b*^ homozygotes had slightly raised ABR thresholds compared with control littermates from the earliest age studied, 3 weeks old, and thresholds slowly increased with age, especially visible at low and middle stimulus frequencies. High frequencies showed progressive increase in ABR thresholds with age in homozygotes, heterozygotes and wildtypes due to the presence of the *Cdh23*^*ahl*^ allele in the C57BL/6 N genetic background of the strain (Kane et al., 2012; Mianné et al., 2016). DPOAE thresholds at 14 weeks were raised, similar to ABR thresholds, indicating that outer hair cells were not functioning normally in the *Sgms1*^*tm1b*^ homozygotes. A minimal amount of sporadic outer hair cell degeneration was found at 4 weeks old, but this would not be enough to explain the increased ABR thresholds in mutants at this age.

EP was measured at three, four and 14 weeks old and showed a stable reduction of around 30–40 mV compared with wildtype and heterozygous littermates. The reduced EP could explain the raised ABR thresholds at three and four weeks old but not the gradual increase in ABR thresholds observed with age. It is possible that increasing hair cell damage or dysfunction may underlie the progressive increase in ABR thresholds up to 14 weeks or older. The appearance of the marginal cell surfaces in whole mount preparations of the stria was more severely affected in mutants at 14 weeks old than at 3 and 4 weeks old, but EP measured from the basal turn remained consistent over this time. Thus, the link between the severity of morphological changes of marginal cells and EP level is not clear.

*Sgms1* expression was assessed by a LacZ reporter assay and immunohistochemistry, and strong expression was seen in the marginal cell layer of the stria vascularis, corresponding to the disorganisation of marginal cell surfaces and variable Kcnq1 labelling of marginal cells seen in whole mount preparations of mutants. Kcnj10 expression in intermediate cells appeared comparable in homozygous mutants and wildtypes. These observations combined with the early reduction in EP suggest that the marginal cells may be the primary cell type affected by the lack of Sgms1. Furthermore, previous *in vitro* studies indicated that Sgms1 positively regulates the density of Kcnq1/Kcne1 potassium channels at the cell surface (Wu et al., 2016), further supporting the marginal cells as the likely site of lesion. However, earlier reports also indicate a role for Sgms1 in the immune system (Toshima et al., 2019; Wang et al., 2019; Li et al., 2012), in vascular boundaries (Wittmann et al., 2016) and in oxidative stress (Yano et al., 2011, 2013) suggesting alternative hypotheses for the mechanism of the cochlear pathology in *Sgms1*^*tm1b*^ mutant mice.

Our analysis replicated most of the key findings reported in an independent *Sgms1* mouse mutant which deleted the first coding exon of the gene (Yano et al., 2011; Lu et al., 2012). Both studies found that homozygous mutants were subviable; ABR thresholds increased gradually with age especially affecting lower frequencies; DPOAE thresholds were increased; EP was reduced by around 30–40 mV in mutants; and strial marginal cell boundaries were disorganised in mutants along the length of the cochlear duct. Both studies found a patchy reduction in expression of Kcnq1, a key molecule in marginal cell function. In contrast to our finding of a stable reduction in EP, the earlier report suggested a progressive reduction in EP, because their recordings in one month old mutants were reduced by around 20 mV followed by around 35 mV at 3 and 6 months old. Lu et al. (2012) additionally measured potassium levels in scala media endolymph and found them to be normal, and also reported that there was only limited degeneration of hair cells up to 6 months old in the homozygotes. Both studies support the suggestion that hair cell degeneration is limited and secondary to a primary endocochlear potential deficit, but the reason for the progressive increase in ABR thresholds up to 6 months old in both mutants is not clear.

The *Sgms1*^*tm1b*^ homozygous males were infertile. Deafness associated with male infertility has been described in humans, and most of these cases appear to be due to a contiguous gene deletion of both *STRC* (leading to deafness) and *CATSPER2* (leading to infertility) (Zhang et al., 2007). The link between male infertility and hearing impairment in the *Sgms1*^*tm1b*^ homozygous males is unclear, but it is of interest that in a large-scale phenotypic screen of newly-generated mutants, of the 38 new genes identified as underlying deafness, six also showed male infertility (*Pex3, Mkrn2, Herc1, Camsap3, Mcph1, Usp42*; Ingham et al., 2019). These were not due to contiguous gene deletions, suggesting an underlying common pathway, but there is no obvious link between these seven genes.

*Sgms1* is the third gene involved in the S1P signalling pathway where progressive hearing loss is associated with reduced EP, along with *Spns2* and *S1pr2* (Lu et al., 2012; Ingham et al., 2019; Chen et al., 2014; Ingham et al., 2016). However, there may be different cell types involved in the primary pathology in the three different mutants judging from the expression patterns of other key strial cell markers; for example, Kcnj10 showed reduced expression in *Spns2* mouse mutants while this was normal in the *Sgms1* homozygous mutants. All three genes have been associated with auditory function in the human population, shown by association between genomic markers within or close to each gene and audiometric thresholds at 1 or 4 kHz (Table 2; Ingham et al., 2016; Ingham et al., 2019). Single gene mutations in *SPNS2* and *S1PR2* have also been shown to underlie childhood deafness in humans (Santos-Cortez et al., 2016; Ingham et al., 2019; Mardani et al., 2023; Hofrichter et al., 2018). It is notable that the hearing loss in *Sgms1*^*tm1b/tm1b*^ mutants starts later and progresses over a much slower time course than in *Spns2*^*tm1a*^ or *S1pr2st*^*df*^ or *S1pr2* knockout mutants. This may make the *Sgms1*^*tm1b*^ allele particularly useful for studies of potential interventions in S1P-related hearing loss, as there is a longer window of opportunity to intervene.

## Supplementary Material

Supplementary data Supplementary material associated with this article can be found, in the online version, at doi:10.1016/j.heares.2024.109091.

## Figures and Tables

**Table 1:** Primer sequences (5′ to 3′) for genotyping *Sgms1*^*tm1a*^ and *Sgms1*^*tm1b*^ alleles.

Reaction name	Forward primer	Reverse primer	Size of band (bp)	Interpretation
Sgms1_wildtype	CTGCCTGTCTATTCCTGCCC	ATGGGGCATCGCAGACTAAC	487	Wildtype *Sgms1* allele present
Sgms1_cassette	CTGCCTGTCTATTCCTGCCC	TCGTGGTATCGTTATGCGCC	195	tm1a or tm1b allele present at *Sgms1* locus
tmlb	CGGTCGCTACCATTACCAGT	ACTGATGGCGAGCTCAGACC	380	tm1b version present

**Table 2:** Associations between genomic markers close to or within *SPNS2, S1PR2* and *SGMS1* and audiometric thresholds in people in the 1958 British Birth Cohort. Significant P values are in bold; *p*<0.0083 after correction for multiple testing. Data for *SPNS2* and *S1PR2* were previously published in Ingham et al. (2016, 2019).

	Thresholds at 1kHz			Thresholds at 4kHz	
	P value	Peak marker		P value	Peak marker
*SPNS2*	0.034	17:4,444,238:G_G		**0.000204**	rs117002379
*S1PR2*	**0.001644**	rs74930654		**0.001105**	19:10,441,093
*SGMS1*	**0.001725**	rs138982939		**0.000475**	rs139282282

## Data Availability

Mouse data presented are available from the authors on request. Mouse mutants are available via the public repository European Mouse Mutant Archive.

## Abbreviations

S1P: sphingosine-1-phosphate
ABR: Auditory Brainstem Response
DPOAE: Distortion Product Otoacoustic Emissions.
