# Supplementary material for: A new mutation of Sgms1 causes gradual hearing loss associated with a reduced endocochlear potential
Source: Hear Res. Author manuscript; Available in PMC 2026 Jun 1. (PMC7619117; doi:10.1016/j.heares.2024.109091)
Supplement: Supplementary data — Supplementary material associated with this article can be found, in the online version, at doi:10.1016/j.heares.2024.109091. [file EMS213888-supplement-Supplementary_data.pdf]

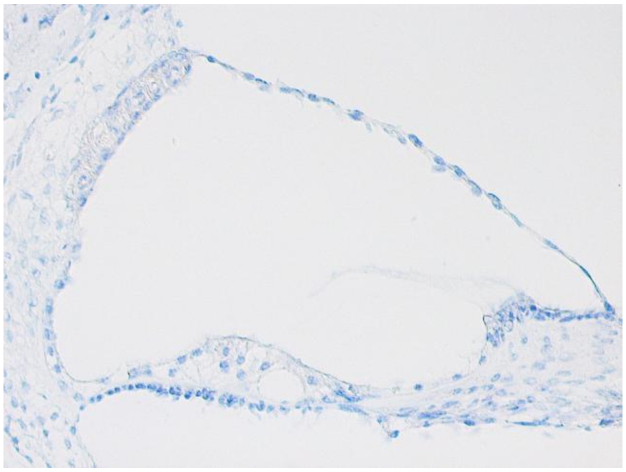

Supplementary figure 1: Negative control for immunohistochemistry. The negative controls for immunohistochemistry were obtained by omitting the anti-Sgms1 antibody and only adding the secondary antibody (anti-rabbit IgG). No brown staining was observed in the cochlea. Cochlear duct of a wildtype mouse at P14.

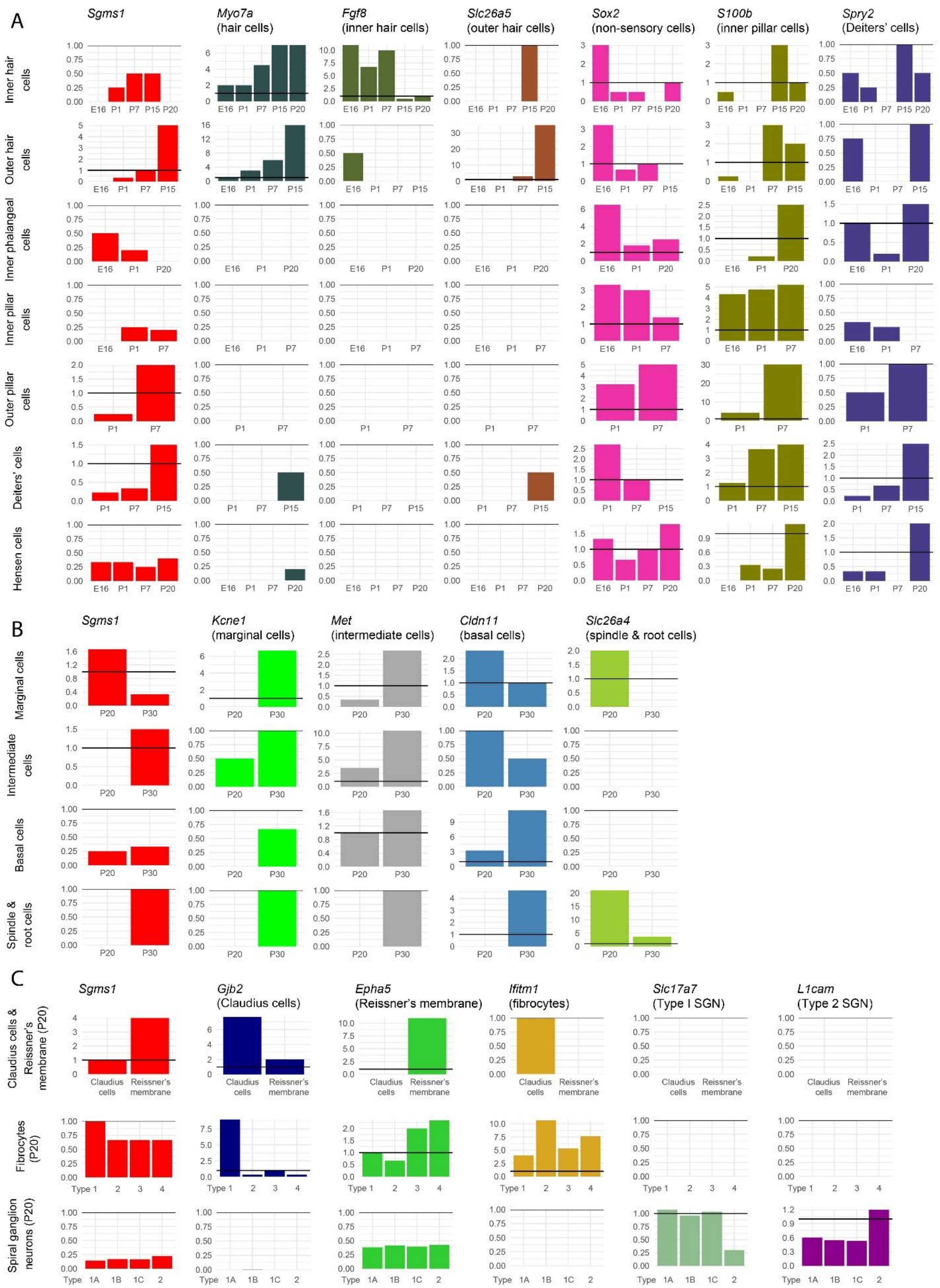
A
Sgms1
Myo7a
(hair cells)
Fgf8
(inner hair cells)
Slc26a5
(outer hair cells)
Sox2
(non-sensory cells)
S100b
(inner pillar cells)
Spry2
(Deiters' cells)
Inner hair cells
Outer hair cells
Inner phalangeal cells
Inner pillar cells
Outer pillar cells
Deiters' cells
Hensen cells
E16
P1
P7
P15
P20
B
Sgms1
Kcne1
(marginal cells)
Met
(intermediate cells)
Cldn11
(basal cells)
Slc26a4
(spindle & root cells)
Marginal cells
Intermediate cells
Basal cells
Spindle & root cells
P20
P30
C
Sgms1
Gjb2
(Claudius cells)
Epha5
(Reissner's membrane)
Ifitm1
(fibrocytes)
Slc17a7
(Type I SGN)
L1cam
(Type 2 SGN)
Claudius cells & Reissner's membrane (P20)
Fibrocytes (P20)
Spiral ganglion neurons (P20)
Claudius cells
Reissner's membrane
Type 1
2
3
4
Type
1A
1B
1C
2

Supplementary figure 2: Expression of candidate genes in different cochlear cell types. Gene expression in the mouse inner ear was assessed using single cell RNAseq data obtained from the gEAR portal (https://umgear.org, accessed December 2021; Orvis et al. 2021). **A** Expression of *Sgms1* compared to known marker genes at stages from E16 to P15 in the mouse organ of Corti, normalised to *Hprt*. **B** Expression of *Sgms1* compared to known marker genes at P20 and P30 in the mouse lateral wall, normalised to *Hprt*. **C** Expression of *Sgms1* compared to known marker genes for cell types where data were only available at P20. Cell types were defined by the experiment from which the data originated. The horizontal line at y=1 on each plot indicates the *Hprt* expression level. Marker genes: *Myo7a* (hair cells), *Fgf8* (inner hair cells), *Slc26a5* (outer hair cells), *Sox2* (non-sensory cells), *S100b* (inner pillar cells), *Spry2* (Deiters' cells), *Kcne1* (marginal cells), *Met* (intermediate cells), *Cldn11* (basal cells), *Slc26a4* (spindle and root cells), *Gjb2* (Claudius cells), *Epha5* (Reissner's membrane), *Ifitm1* (fibrocytes), *Slc17a7* (type 1 spiral ganglion neurons (SGN)), *L1cam* (type 2 SGN). Original data from Kolla et al. 2020; Ranum et al. 2019; Xue et al. 2021; Korrapati et al. 2019; Shrestha et al. 2018; Petitpre et al. 2018.

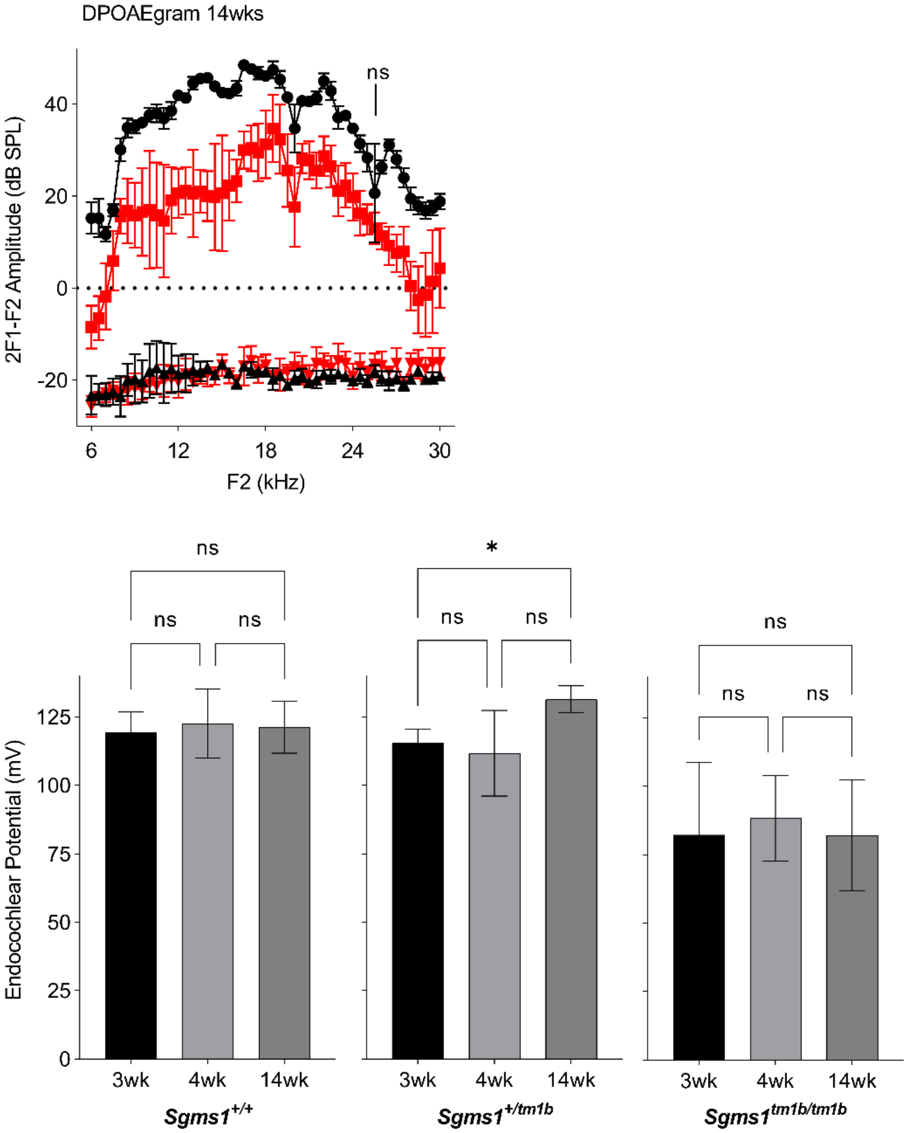

Supplementary figure 3: DPOAEgram and age-related changes in EP.

Top: In addition to measuring DPOAE thresholds in the mice aged 14 weeks old, we also measured DPOAEgrams, using a fixed F2 level of 70dB SPL with F2 frequencies ranging from 6000 Hz to 30000 Hz, in 500 Hz steps. For each F2 stimulus, we measured the evoked 2F1-F2 DPOAE amplitude and associated noisefloor. These are plotted for *Sgms1*$^{tm1b/tm1b}$ and *Sgms1*$^{+/+}$ mice, in red and black, respectively. (The lower curve at approximately -20 dB SPL represents the noisefloor of the measurement.) The amplitude of the DPOAE was significantly reduced in the mutant mice for all F2 test frequencies except for 25500 Hz (indicated by “ns”), when compared using multiple Mann-Whitney tests of the non-parametric data ($p < 0.05$).

Bottom: Endocochlear potential was compared across age (from 3- 14 weeks) for each genotype. Kruskall-Wallis ANOVA, with Dunn's multiple comparisons tests, showed there were no age-related declines in EP for any genotype ($p > 0.05$).
